# TLR3 and TLR7 Modulate IgE Production in Antigen Induced Pulmonary Inflammation via Influencing IL-4 Expression in Immune Organs

**DOI:** 10.1371/journal.pone.0017252

**Published:** 2011-02-25

**Authors:** Liesu Meng, Xiaojing He, Wenhua Zhu, Xudong Yang, Congshan Jiang, Qingzhu Sun, Asim Raza M.B., Simeng Zhang, Qian Xue, Xinfang Xie, Shemin Lu

**Affiliations:** 1 Department of Genetics and Molecular Biology, Xi'an Jiaotong University School of Medicine, Xi'an, Shaanxi, China; 2 Key Laboratory of Environment and Genes Related to Diseases, Xi'an Jiaotong University, Ministry of Education, Xi'an, Shaanxi, China; Oregon Health and Science University, United States of America

## Abstract

**Background:**

Toll-like receptors (TLRs) as pattern recognition receptors, participate in both innate and adaptive immune responses, and seem to play an important role in the pathogenesis of asthma. This study aimed to identify key TLRs involved in antigen induced pulmonary inflammation (AIPI), a rat model for asthma, and to explore the role of TLRs in the disease development.

**Methods and Findings:**

E3 rats were sensitized with ovalbumin (OVA)/alum intraperitoneally and intranasally challenged with OVA to induce AIPI model. TLR1-9 and cytokine mRNA expression in spleen, lung and mediastinal lymph node (mLN) tissues were screened by quantitative real-time polymerase chain reaction. TLR7 expression was found to be significantly down-regulated in spleen while TLR3 and TLR8 expression was up-regulated in mLN of AIPI rats. Furthermore, imiquimod (a ligand of TLR7) and TLR3 specific short-hairpin RNA plasmid for RNA interference were administrated, respectively, *in vivo* to AIPI rats to observe their effects on the disease by assessing various asthmatic parameters. The numbers of total cells, eosinophils, macrophages and lymphocytes were counted according to differential morphology in bronchoalveolar lavage fluid. Serum IgE and OVA specific IgG_1_ concentration was detected by enzyme-linked immunosorbent assay. The results showed that both TLR7 ligand treatment and TLR3 RNAi *in vivo* decreased serum IgE level and interleukin-4 mRNA expression.

**Conclusion/Significance:**

TLR3 in mLN and TLR7 in spleen both systemically modulate disease development in AIPI rats via altering serum IgE concentration relevant to Th2 responses. And these findings may provide an important clue for further research in the asthma pathogenesis and suggest a new remedy for asthma treatment.

## Introduction

Asthma with a high mobidity is an inflammatory disease of the lower airways. It is widely accepted that Th2 cells are critical regulators of asthma pathogenesis [1], [2]. Th2 cells secrete inflammatory cytokines, such as interleukin-4 (IL-4), and interleukin-5, which play key roles in the development of asthma [3]. However, the pathogenesis of asthma is so complex that it is still not fully understood up to now. Recently many researchers focus on toll-like receptors (TLRs) which have been considered to be important factors in the development of asthma. TLRs, which play a crucial role in both innate and adaptive immune response, can recognize pathogen-associated molecular patterns and their activation can induce the production of proinflammatory cytokines [4]. TLRs are widely expressed in the cells of the respiratory system, such as airway epithelial cells, airway smooth muscle cells, mast cells, fibroblasts and tracheal smooth muscle cells [5].

Association studies in asthma patients have demonstrated that the close relations between gene polymorphisms of TLRs and asthma [6]–[10]. Besides, animal models have been used to explore the roles of TLRs in asthma. TLR2 is reported to play a pro-allergic role in the allergic host, and stimulation of TLR2 ligand augments Th2 responses and exacerbates airway hyperresponsiveness (AHR) [11]–[13]. However, it is also reported that a synthetic TLR2 ligand reduces eosinophil count in the bronchoalveolar lavage fluid (BALF), AHR as well as serum IgE level [14]. dsRNA triggers the exacerbation of the pulmonary allergic reaction through TLR3/TRIF-dependent pathway [15]. Interestingly, it has been found that both low and high doses of poly(I:C) could induce pulmonary inflammation, and lung inflammation enhanced by low-dose dsRNA depended on Th2 immune response, whereas lung inflammation by high-dose dsRNA depended on Th1 immune response [16]. Although the role of TLR4 in asthma development is controversial, growing evidence proves that high levels of LPS exert beneficial effect in asthma models due to the induction of a Th1 response while low dose of LPS biases the immune response toward a Th2 phenotype and results in aggravation of experimental asthma [17], [18]. Some researchers have demonstrated that synthetic TLR7 ligands can inhibit Th2 responses, airway remodeling and attenuate airway inflammation in mouse asthma models [19]–[21]. TLR9 activation can down-regulate allergic responses by promoting Th1 cytokine generation and synthetic TLR9 agonists have been developed as new drugs to treat asthma [22].

Although there have already been lots of studies implying the role of TLRs in asthma, few studies have characterized the expression profile of TLRs in the animal asthma model, and the roles of TLRs are still controversial and obscure. So in the present study we constructed antigen induced pulmonary inflammation (AIPI) model in E3 rats and tried to identify the key TLRs in the disease development by detecting TLR1-9 expression in spleen, lymph nodes and lung. The results indicated TLR3 in spleen was up-regulated while TLR7 in mediastinal lymph node (mLN) was down-regulated in AIPI model. And intervention of TLR3 and TLR7 by RNA interference (RNAi) or ligand stimulation influenced IgE production and IL-4 expression. All these findings illustrate that TLR3 and TLR7 in immune organs play a vital role in the development of asthma.

## Results

### TLR3, TLR7 and TLR8 mRNA expression was regulated in immune organs of E3 rats in AIPI model

**Figure 1:**
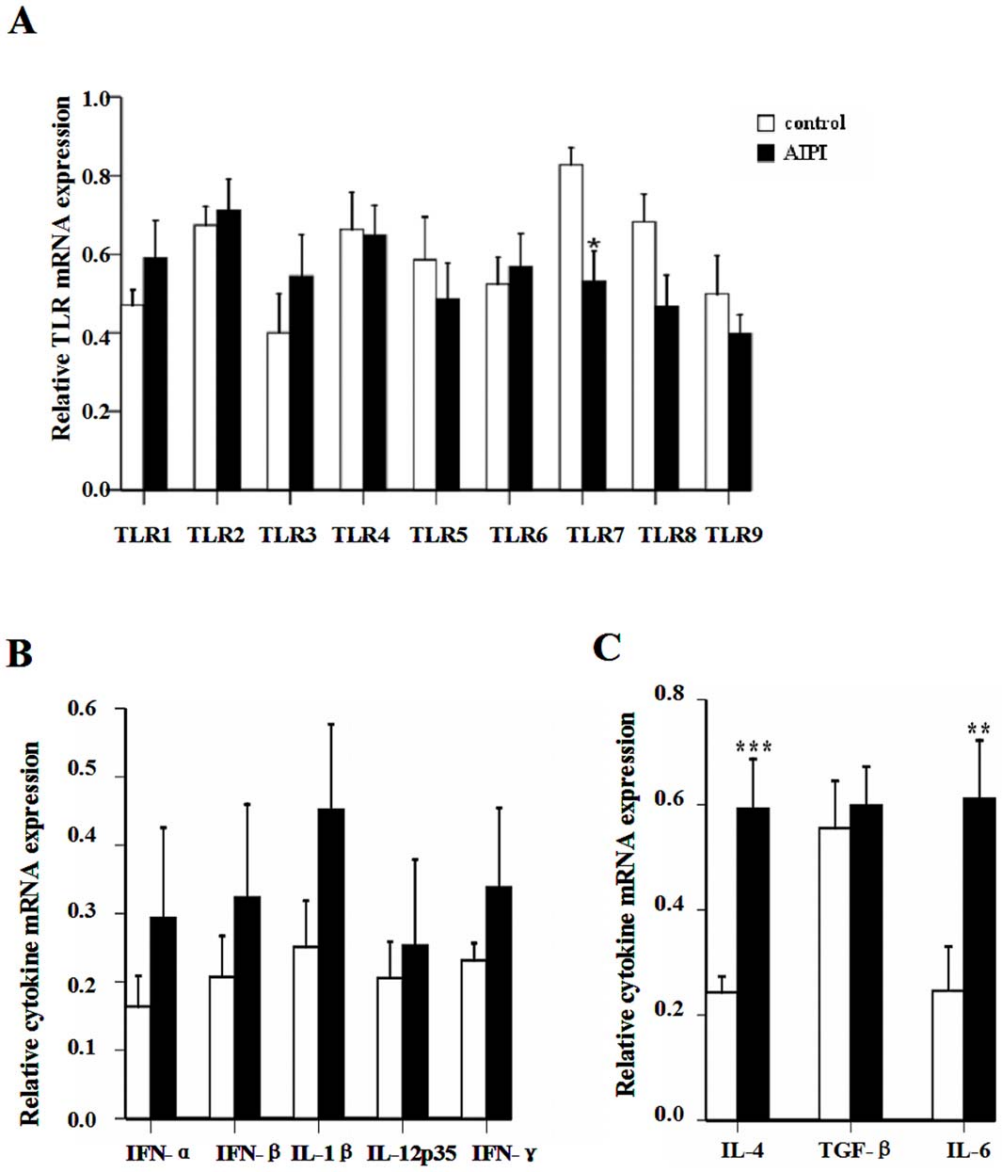
TLR and cytokine mRNA expression in spleen of rats from control group and AIPI group. TLR1-9 mRNA expression (A), IFN-α, IFN-β, IL-1β, IL-12p35, IFN-γ mRNA expression (B) and IL-4, TGF-β, IL-6 mRNA expression (C) were measured by QPCR. The rats in AIPI group were sensitized and challenged with OVA, while the rats of control group was administrated PBS. Results show as mean ±SEM from 10 rats in each group. *P<0.05, **P<0.01 and ***P<0.001; significantly different from control group.

To investigate the role of TLRs in asthma development, we constructed AIPI model by using ovalbumin (OVA) to sensitize and challenge E3 rats and detected TLR1-9 mRNA expression in spleen, mLN and lung of the rats by using quantitative real-time polymerase chain reaction (PCR). Various asthmatic parameters were assessed to confirm the success of the model adopted in the present study [23]. TLR7 transcripts in spleen tissues from AIPI rats were significantly reduced compared with control rats, and no significant difference in other TLR mRNA expression levels was detected (Figure 1A). Besides, we examined the expression of relative cytokines in spleen tissues by QPCR (Figure 1B and C). Significantly elevated levels of IL-4 (*P*<0.001) and interleukin-6 (IL-6) (*P*<0.01) mRNA expression were observed in the AIPI rats. However, OVA treatment did not change the levels of transforming growth factors-β (TGF-β) mRNA expression in the spleen tissues.

**Figure 2:**
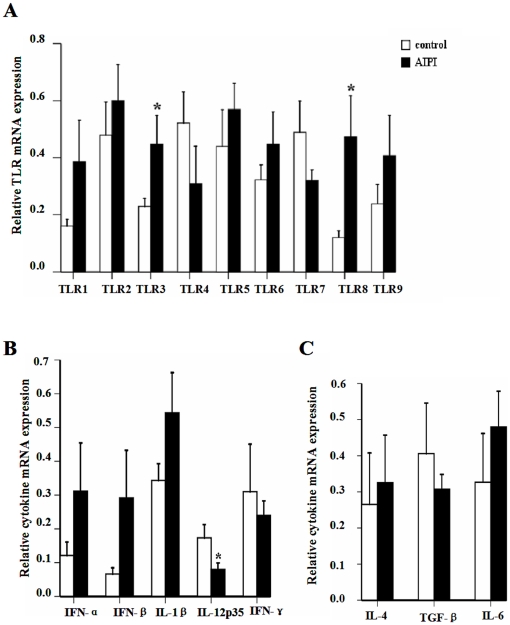
TLR and cytokine mRNA expression in mLN of rats from control group and AIPI group. TLR1-9 mRNA expression (A), IFN-α, IFN-β, IL-1β, IL-12p35, IFN-γ mRNA expression (B) and IL-4, TGF-β, IL-6 mRNA expression (C) were measured by QPCR. The rats in AIPI group were sensitized and challenged with OVA, while the rats of control group was administrated PBS. Results show as mean ±SEM from 10 rats in each group. *P<0.05; significantly different from control group.

In addition, we also measured TLR1-9 and cytokine mRNA expression in mLN tissues from AIPI and control (Figure 2). Using QPCR, we observed that AIPI rats expressed higher TLR3 and TLR8 mRNA compared to control group (Figure 2A). And outstandingly, interleukin-12p35 transcripts were decreased in the mLN tissues of AIPI rats (Figure 2B).

**Figure 3:**
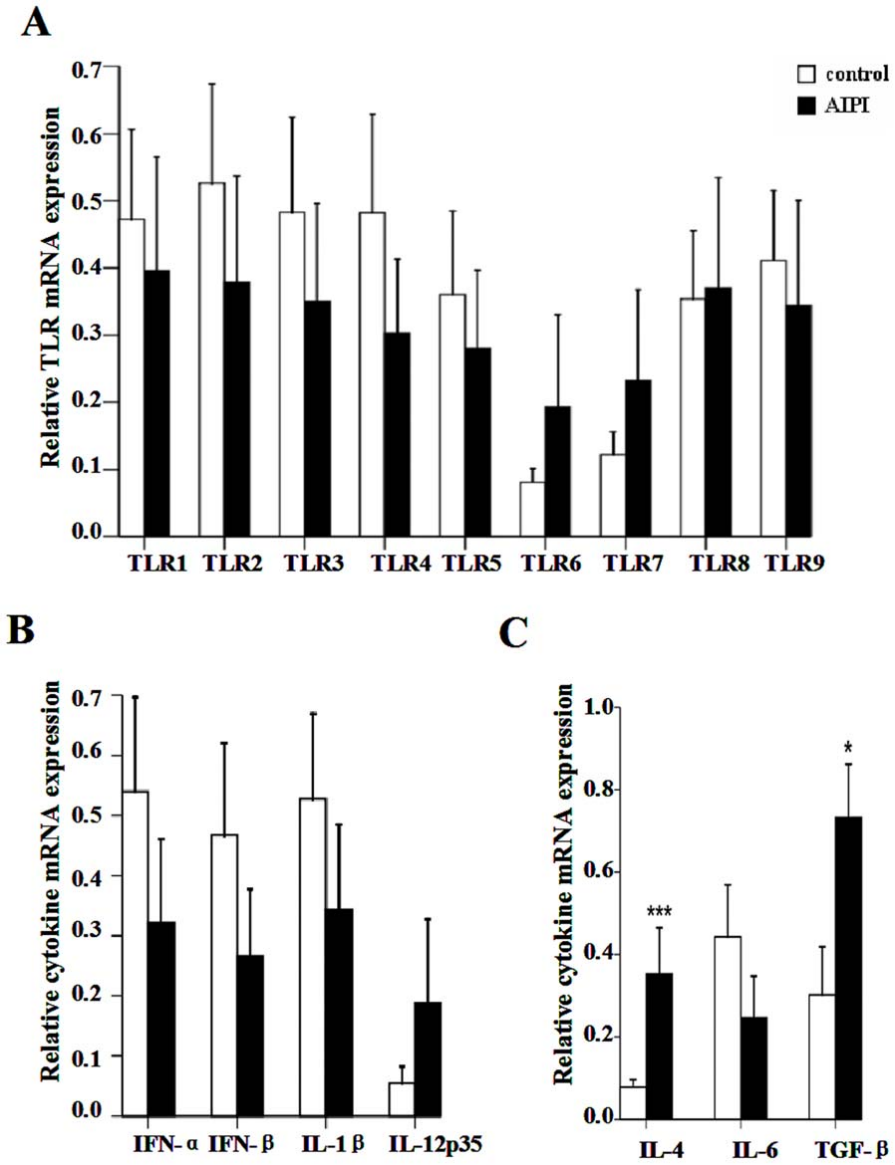
TLR and cytokine mRNA expression in lung of rats from control group and AIPI group. TLR1-9 mRNA expression (A), IFN-α, IFN-β, IL-1β, IL-12p35 mRNA expression (B) and IL-4, TGF-β, IL-6 mRNA expression (C) were measured by QPCR. The rats in AIPI group were sensitized and challenged with OVA, while the rats of control group was administrated PBS. Results show as mean ±SEM from 10 rats in each group. *P<0.05; ***P<0.001; significantly different from control group.

After investigating TLR1-9 mRNA expression in the lung tissues, we did not found any significant difference of TLR mRNA expression level in the lungs between AIPI and control groups (Figure 3A). And cytokine mRNA expression showed that IL-4 (*P*<0.001) and TGF-β transcripts were significantly increased in the lung tissues from AIPI rats (Figure 3C).

### TLR7 agonist administration has marked effect on AIPI via influencing serum IgE level

**Figure 4:**
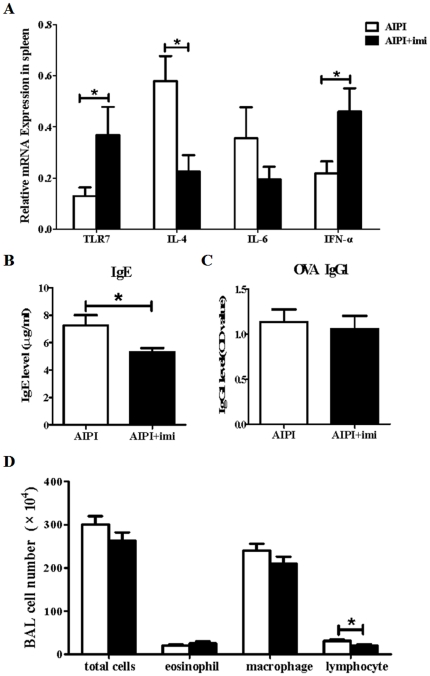
Assessment of various asthmatic parameters in TLR7 agonist stimulated AIPI rats. E3 rats were injected intraperitoneally with vehicle or TLR7 agonist on day 13 and 17 during the period of AIPI induction. The rats of AIPI group (n = 9) received 1 ml saline and the rats of AIPI+imi (imiquimod) group (n = 9) received 100 µg miquimod. TLR7 mRNA and cytokine mRNA expression levels in spleen were measured by QPCR (A). Levels of serum total IgE (B) and OVA-specific IgG1 (C) were measured by ELISA. Total cell numbers in BALF were counted under light microscope using a hemocytometer and values of macrophage, eosinophil and lymphocyte counts in BALF were expressed as the percentage of cell numbers of total cells (D). Results show as mean ±SEM from 9 rats in each group. *P<0.05; **P<0.01; ***P<0.001; significantly different from AIPI group.

To further determine the role of down-regulated TLR7 on asthma development, we investigated whether TLR7 agonist could affect AIPI development. E3 rats were treated with vehicle or imiquimod at indicated time points during AIPI induction, respectively. And various asthmatic parameters were assessed. First we detected TLR7, IL-4, IL-6 and interferon-α (IFN-α) mRNA expression in spleen using QPCR. TLR7 and IFN-α transcripts in spleen tissues were significantly increased after the treatment with imiquimod, while IL-4 mRNA level was markedly decreased (Figure 4A). Furthermore, it was obviously showed that a significant difference was detected in the level of IgE production between groups treated with vehicle and imiquimod (Figure 4B). But imiquimod did not influence OVA-specific IgG_1_ release (Figure 4B and C). Statistical analysis of cell number counted in BALF (Figure 4D) showed that imiquimod administration only influenced lymphocyte recruitment to the lung. The numbers of total cells, eosinophils and macrophages were unaffected. Taken together, these findings demonstrated that TLR7 agonist mainly influenced serum IgE release and hardly affected other asthmatic parameters.

### TLR3 RNAi in vivo attenuated AIPI through suppressing IgE release

**Figure 5:**
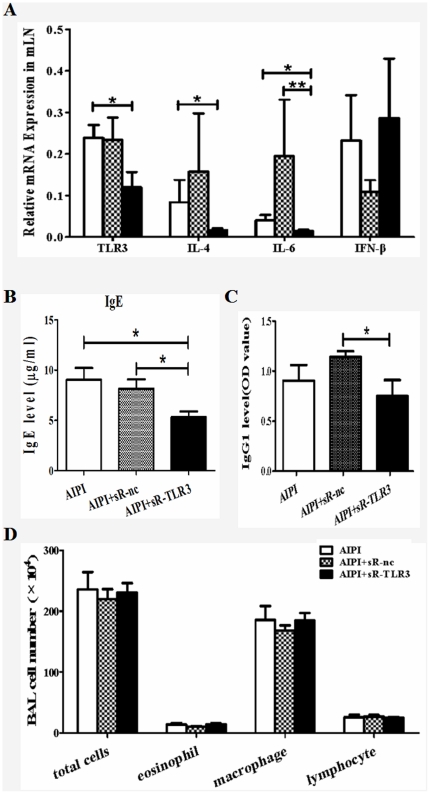
Assessment of various asthmatic parameters in TLR3 RNAi AIPI model. E3 rats were intranasally instilled with either saline or plasmids on day 13 and 17 during the period of AIPI induction. The rats of AIPI group (n = 9) received 50 µl saline, the rats of AIPI+sR-nc (n = 9) group received 50 µg shRNA-nc plasmid and the rats of AIPI+sR-TLR3 group (n = 9) received 50 µg shRNA-TLR3 plasmid. TLR3 mRNA and cytokine mRNA expression levels in mLN were measured by QPCR (A). Levels of serum total IgE (B) and OVA-specific IgG1 (C) were measured by ELISA. Total cell numbers in BALF were counted under light microscope using a hemocytometer and values of macrophage, eosinophil and lymphocyte counts in BALF were expressed as the percentage of cell numbers of total cells (D). Results show as mean ±SEM from 9 rats in each group. *P<0.05 or **P<0.01 or ***P<0.001 denotes significance between two groups.

To confirm that up-regulated TLR3 played an important part in asthma development, we interfered TLR3 expression with specific short-hairpin RNA (shRNA) plasmid. The rats were intranasally instilled with saline, shRNA-nc plasmids or shRNA-TLR3 plasmids at indicated time points during AIPI induction, accordingly. And various asthmatic parameters were assessed. We first examined TLR3 mRNA expression in rats to evaluate the efficiency of RNA interference. TLR3 mRNA level in mLN of AIPI+sR-TLR3 group was significantly lower than that of AIPI group (*P*<0.05), which demonstrated the interfering effect of shRNA on TLR3 mRNA expression (Figure 5A). Besides, we noticed that shRNA-TLR3 plasmid administration considerably down-regulated IL-4 and IL-6 mRNA expression in mLN tissues (Figure 5A). Serum IgE level was markedly lower in AIPI+sR-TLR3 group, compared to AIPI group and AIPI+sR-nc group (Figure 5B). Additionally, TLR3 RNAi also significantly inhibited OVA-specific IgG_1_ release compared to shRNA-nc plasmids administration (Figure 5C). Furthermore, we counted total cell, eosinophil, macrophage and lymphocyte number in BALF. However, no significant difference was detected among the three groups (Figure 5D). Taken together, these data suggested that administration of TLR3 RNAi to AIPI rats influenced some parameters and alternation of serum IgE level was the most obvious.

## Discussion

Allergic asthma is considered to be an immune disease, in which Th2 cells are critical regulators. TLRs play a vital role in T cell polarization while spleen and lymphonodes are main lymphatic organs in which Th0 cells differentiate to Th1 or Th2 cells. Therefore the present study focuses on TLRs in spleen and mLN, the main draining lymphonode of lung and bronchus, related with asthma. With the limitation to collect patients' material, most researches elucidating the relationship between TLRs and asthma have been conducted with asthma animal models [24]–[27]. Thus in the present study, we adopted AIPI model to explore the expression profile of TLRs and evaluate TLR expression regulation in spleen, mLN and lung.

The AIPI model is established with E3 rats, an inbred rat which is susceptible to asthma but resistant to Th1 type diseases. The model shows more similar phenotypes to human asthma by histological and biochemical estimation compared to other rat strains [23], [28]–[31]. By assaying QPCR, we screened the TLR expression and found that TLR7 expression was significantly down-regulated in spleen while TLR3 and TLR8 expression was up-regulated in mLN in AIPI rats. Although TLRs are expressed on numerous types of cells in respiratory system, in the present study TLR mRNA expression in lung did not show difference in two groups while IL-4 and TGF-β expression increased significantly in AIPI rats. While researchers mainly focus on TLRs in lung and airway, the present results lead us to pay more attention to the TLRs in immune organs, such as spleen and mLN, which may play a crucial role in the pathogenesis of asthma. So next we investigated the role of TLR7 in spleen and TLR3 in mLN on the disease development further.

Down-regulated TLR7 expression in spleen may reflect a systemic immune regulation of AIPI rats. In fact, several studies have reported that TLR7 agonists could attenuate the symptom of experimental asthma and virus-induced airway dysfunction [19]. And a recent study has showed that imiquimod significantly inhibits chronic inflammation, persistent AHR and airway remodeling in chronic experimental asthma [20]. Activation of TLR7 can initiate a very strong Th1 response through inducing NF-κB signaling. This effect might be performed by modulating key master switches GATA-3 and T-bet that results in committing T helper cells to a Th1 phenotype [32]. After imiquimod intraperitoneal injection to AIPI rats, TLR7 mRNA and IFN-α mRNA levels both elevate in the spleen tissues while IL-4 mRNA level decreases, which is consistent with the effect of imiquimod in anticipation. Previous studies showed that imiquimod application could reduce IL-4 in BALF and serum [20], [32]. And it was found that resiquimod treatment would result in the decrease of Th2 cytokines, such as IL-4 and interleukin-5 mRNA and protein in lung of mice or rat model [33]–[35]. Our present results indicated that notably in spleen, the central peripheral immune organ, the induction of TLR7 also inhibited the production of Th2 cytokines. Furthermore, in our results, imiquimod also modulated IgE production just as previous studies showed [20], [33], [35], [36]. Decreased IgE has been shown in isolated human B cells and allergic human B cells treated with resiquimod [37], [38]. IgE is a characteristic biomarker of asthma patients. The allergen-activated Th2 cells produce IL-4, which stimulates B cells to undergo heavy-chain class switching from IgM to IgE [3], [39]. Hence TLR7 in spleen may play a key role in the development of asthma via IL-4 to induce the production of allergen-specific IgE by B cells. However, in our model imiquimod failed to further alter other asthmatic parameters, such as eosinophils number in BALF, which was opposite to other studies before [32], [33], [35]. These findings suggest that spleen TLR7 is involved into only an aspect of the pathogenesis of AIPI and mainly affect systemic immune response just like IgE level. Furthermore, it was found that imiquimod could interact with adenosine receptor as an antagonist, and appeared to suppress the cAMP production and subsequently induce the proinflammatory cytokines via activating the NF-κB pathway [40]. In the present study, imiquimod increased the IFN-α expression in the spleen of AIPI rats which should be mediated by IRF-7 activation and seem specific for TLR7 signaling. So we believe that imiquimod might play its role mainly by TLR7 signaling pathway, although we still could not rule out the involvement of the adenosine receptors by now.

Up-regulated TLR3 expression in mLN, the draining lymphonode of lung tissue may reflect a local immune response of AIPI rats. It is reported that Rhinoviruses, a major etiology of the common cold, could increase the expression of TLR3 on the human bronchial epithelial cells, and trigger exacerbation of the pulmonary allergic reaction through TLR3/TRIF-dependent or TLR3/IRF3-dependent pathway [15], [41], [42]. Such studies imply that TLR3 may play a critical role in asthma. Nevertheless, a recent study has been reported that application of TLR2, TLR3, TLR4 and TLR7 agonists all show a protective effect for asthma [43]. And TLR3-induced IL-6 is similar in asthmatic and control subjects while TLR7 function is reduced in adolescents [44]. However, long-term activation of TLR3 induces inflammation and impaired lung function in mice [45]. Therefore the role of TLR3 in asthma is still obscure for researchers. Based on the up-regulation of TLR3 mRNA level in mLN of AIPI rats, we knockdowned TLR3 expression of AIPI rats through *in vivo* RNAi. Jeon et al. found that AHR enhanced by low or high doses of poly(I:C) did not develop in TLR3-deficient mice, while OVA-specific IgE production enhanced by low-dose poly(I:C) and serum OVA-specific IgG2a levels enhanced by high-dose poly(I:C) also did not develop in TLR3-deficient mice [16]. The lung inflammation were induced both by low-dose and high-dose poly(I:C), but the former played its role by Th2 immune response and the latter by Th1 response [16]. In the present study IL-4 mRNA in mLN, IgE and IgG1 levels in serum were reduced by TLR3 RNAi systemically. It might hint us that TLR3 may exert it function by Th2 immune response, but Th1 response could not be excluded. Furthermore, TLR3-shRNA did not alter total cell number and macrophage in BALF as that shown in other studies, so the pathway by which TLR3 affects IgE level should be under investigation further. Moreover, the effect of TLR3 knockdown suggests the roles of an endogenous TLR3 ligand or constitutive TLR3 activity in AIPI rats, which should be paid more attention to in further study.

In conclusion, TLR3 and TLR7 levels were not modulated in the lung following AIPI, but TLR7 was down-regulated in spleen, while TLR3 was up-regulated in draining lymph nodes. Down-regulation of TLR3 expression and activation of TLR7 both caused decrease of serum IgE level and reduction of IL-4 mRNA expression in immune organs from AIPI rats. The results present in this study suggested that TLR3 and TLR7 in immune organs both systemically modulated disease development in AIPI rats by reducing serum IgE release via IL-4 down-regulation, and which may provide a vital clue for further research in the asthma pathogenesis and suggest a new target for asthma treatment.

## Materials and Methods

### Rats

E3 rats were maintained in a specific-pathogen free facility of Xi'an Jiaotong University School of Medicine. Animal study protocols were approved by the Institutional Animal Ethics Committee of Xi'an Jiaotong University (permission No. 2009-12).

### Induction of AIPI model with ovalbumin

**Figure 6:**
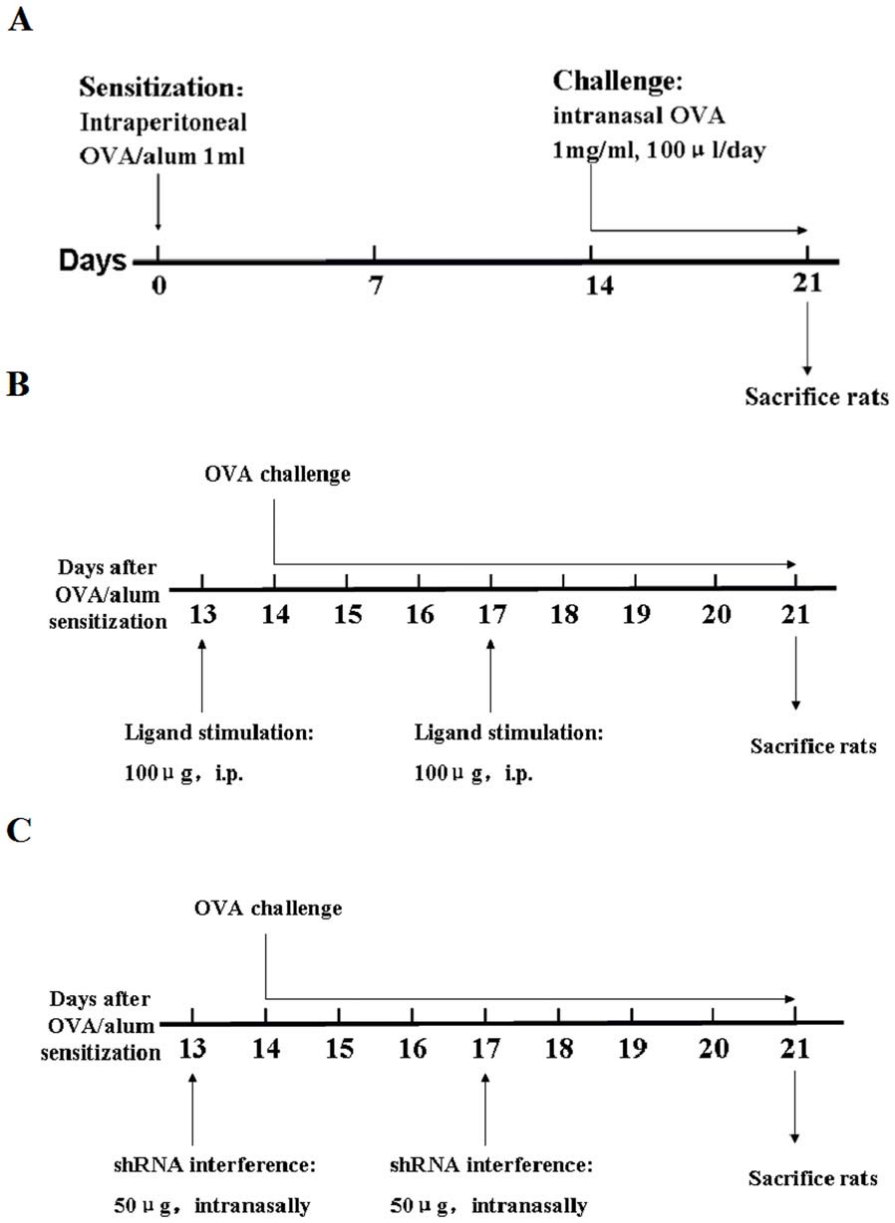
Schematic diagrams of the induction of rat models. Schematic diagrams showed the induction of AIPI (A), TLR7 ligand stimulation (B) and *in vivo* TLR3 RNAi (C) in E3 rats.

Induction of AIPI was performed as follows: the rats were sensitized on day 0 by intraperitoneal injection of 1 mg of OVA (Sigma-Aldrich, St. Louis, MO, USA) emulsified in 50 mg Alum (Sigma-Aldrich, St. Louis, MO, USA) in a total volume of 1 ml PBS; on day 14 the rats were subjected to intranasal challenge of 100 µl OVA/PBS (1 mg/ml) once daily for 7 days, while control group was administrated only PBS for sensitization and challenge (Figure 6A).

### TLR7 agonist stimulation

Eighteen E3 rats, 8–12 weeks of age were randomly distributed into 2 groups that received intraperitoneal injection on day 13 and 17 during the period of AIPI induction as follows: 1 ml saline, served as AIPI group; 100 µg of imiquimod, a TLR7 agonist (Merck, Darmstadt, Germany) in 1 ml saline, named as AIPI+imiquimod group. On day 21 rats were sacrificed and various parameters were measured (Figure 6B).

### TLR3 RNA interference *in vivo*

The target sequence of TLR3 gene for RNAi is ACC TCG ACC TCA CAG AGA A, and the negative control sequence is TTC TCC GAA CGT GTC ACG T [46]. These different shRNA sequences were inserted into pGCsilencer™ U6/Neo/GFP/RNAi plasmid (Genechem, Shanghai, China). Twenty-seven E3 rats aged 8–12 weeks old were intranasally instilled with either saline or plasmids on day 13 and 17 during the period of AIPI induction. The rats were randomly divided into 3 groups: 50 µl saline, served as AIPI group; 50 µg of shRNA-TLR3 plasmid in 50 µl saline, named as AIPI+sR-TLR3 group; and 50 µg of shRNA-nc plasmid in 50 µl saline, named as AIPI+sR-nc group (Figure 6C). Rats were sacrificed on day 21 and various parameters were measured.

### Cell count in BALF

Soon after the lung was removed, BALF was collected via cannulation of the exposed trachea and gentle flushing of the lungs with 2 ml of PBS for three times (total 6 ml). Total cell numbers were counted under light microscope by using a hemocytometer. To count the number of macrophages, eosinophils and lymphocytes in BALF, cytocentrifuged preparations were fixed on slide and stained with Wright-Giemsa differential staining. The number of differential cells were counted according to differentiated morphology and expressed as a percentage of total cells.

### ELISA for rat immunoglobulin concentration measurement

Total IgE and OVA-specific IgG1 concentration in the serum of rats was measured by enzyme-linked immunosorbent assay methods. Briefly, 50 µl of polyclonal anti-IgE antibody (AbD Serotec, Munich, UK) or OVA diluted in NaHCO_3_ buffer was transferred to enzyme-linked immunosorbent assay plates and incubated overnight at 4°C. The plates were washed three times with 250 µl washing buffer (PBS containing 0.05% Tween-20) and blocked with blocking solution (2% BSA in washing buffer) for 1 h at 37°C. Rat serum diluted 100 times was added and the plates were incubated for 1 h at 37°C. Then horseradish peroxidase-conjugated mouse anti-rat kappa/Lambda chain (1 µg/ml) antibody (AbD Serotec, Munich, UK) was added to detect IgE and IgG_1_. A mixture of H_2_O_2_ and tetramethylbenzidine was used as a substrate. The reaction was stopped by 2 M H_2_SO_4_ and absorbance was measured at 450 nm.

### RNA isolation and real-time quantitative RT-PCR

Total RNA was isolated respectively from spleen, mediastial lymph nodes (mLN) and lung by using TRIzol (Invitrogen Life Technology, Carlsbad, CA, USA). cDNA was synthesized from 5 µg of total RNA by utilizing a reverse transcription kit (RevertAid™, Fermentas Life Science, International INC, Canada) in a final volume of 20 µl. QPCR was then performed using SYBR® *Premix Ex Taq*™ II (TaKaRa, Japan) in iQ5 real-time PCR detection system (Bio-Rad, CA, USA). The following program was used: pre-denaturation at 95°C for 30 s and 40 cycles consisting of denaturation at 95°C for 5 s, annealing at 53–65°C for 20 s, and extension at 72°C for 15 s. The specificity of the amplified PCR products was assessed by a melting curve analysis. The primer sequences for TLRs and cytokines can be found in Table 1. Relative quantitation of gene expression was normalized to GAPDH.

**Table 1:** Primer sequences used for QPCR

Name	Type	Sequence (5′-3′)	Expected length (bp)	Optimal annealing temperature (°C)
TLR1	Forward	CAGCAGCCTCAAGCATGTCTA	82	60
	Reverse	CAGCCCTAAGACAACAATACAATAGAAGA		
TLR2	Forward	CTCCTGTGAACTCCTGTCCTT	74	60
	Reverse	AGCTGTCTGGCCAGTCAAC		
TLR3	Forward	GATTGGCAAGTTATTCGTC	205	54
	Reverse	GCGGAGGCTGTTGTAGG		
TLR4	Forward	GATTGCTCAGACATGGCAGTTTC	135	54
	Reverse	CACTCGAGGTAGGTGTTTCTGCTAA		
TLR5	Forward	GGGCAGCAGAAAGACGGTAT	61	60
	Reverse	CAGGCACCAGCCATCCTTAA		
TLR6	Forward	GGAGCCTTCAGTAGCCTTTC	115	60
	Reverse	GGTTGTTCCCCGCTGTTAT		
TLR7	Forward	GTTTTACGTCTACACAGTAACTCTCTTCA	75	60
	Reverse	TTCCTGGAGGTTGCTCATGTTTT		
TLR8	Forward	GGGGTAACACACCGTCTA	150	60
	Reverse	GTCAAGGCGATTTCCACT		
TLR9	Forward	CCGAAGACCTAGCCAACCT	70	60
	Reverse	TGATCACAGCGACGGCAATT		
IFN-α	Forward	GCAACCCTCCTAGACTCATTCTG	83	65
	Reverse	ACCCCTACCTGCTGCATCAG		
IFN-β	Forward	CTTGGGTGACATCCACGACTAC	92	60
	Reverse	GGCATAGCTGTTGTACTTCTTGTCTT		
IFN-γ	Forward	AGAGCCTCCTCTTGGATATCT	309	65
	Reverse	GCTTCCTTAGGCTAGATTCTGGTG		
IL-1β	Forward	CTGTGACTCGTGGGATGATGAC	322	53
	Reverse	CTTCTTCTTTGGGTATTGTTTGG		
IL-12p35	Forward	AAGACATCACACGGGACAAA	300	60
	Reverse	GATTCAGAGACCGCATTAGC		
IL-6	Forward	AAGAAAGACAAAGCCAGAGTC	263	57
	Reverse	CACAAACTGATATGCTTAGGC		
IL-4	Forward	TGCACCGAGATGTTTGTACCAGA	92	63
	Reverse	TTGCGAAGCACCCTGGAAG		
TGF-β	Forward	GGACTACTACGCCAAAGAAG	294	57
	Reverse	TCAAAAGACAGCCACTCAGG		
GAPDH	Forward	CGGCAAGTTCAACGGCACAG	148	60
	Reverse	GAAGACGCCAGTAGACTCCACGAC		

### Statistical analysis

All data were expressed as mean ± standard error (SEM). Mann-Whitney U test and Krusckal-Wallis H test were performed to test statistically significant differences by using SPSS 13.0 (SPSS software, Chicago, IL, USA) for the type of data without normal distribution or homogeneity of variance. A *P* value of less than 0.05 was considered significant.
